# Genetic Polymorphisms Are Differentially Associated With Affective Outcomes in Adolescents With and Without ADHD

**DOI:** 10.1192/bjo.2022.259

**Published:** 2022-06-20

**Authors:** Tünde É. Welker, Nóra Angyal, Zsófia Nemoda, Bea Pászthy, János Réthelyi, Nora Bunford

**Affiliations:** 1 Doctoral School of Mental Health Sciences, Semmelweis University, Budapest, Hungary; 2 Developmental and Translational Neuroscience Research Group, Institute of Cognitive Neuroscience and Psychology, Research Centre for Natural Sciences, Budapest, Hungary; 3 Semmelweis University, Institute of Biochemistry and Molecular Biology, Department of Molecular Biology, Budapest, Hungary; 4 Semmelweis University, 1st Department of Paediatrics, Budapest, Hungary; 5 Semmelweis University, Department of Psychiatry and Psychotherapy, Budapest, Hungary

## Abstract

**Aims:**

Various genetic polymorphisms have been associated with attention-deficit/hyperactivity disorder (ADHD), and some of these have also been implicated in individual differences in affective processing. Yet, no studies to date have examined the complex interrelations across these genetic polymorphisms, ADHD, and affective processing. Several variables (e.g., age, ethnicity, sex) have been shown to affect whether a given genetic variant confers risk. Our aim was to examine whether relevant genetic variants differentially confer risk for negative affectivity (NA) and/or emotion dysregulation (ED), depending on ADHD status.

**Methods:**

Participants were *n* = 297 adolescents (M_age_=15.30 years; *SD* = 1.06; 60.27% boys) with (*n* = 83) and without (DSM-5) ADHD. ADHD- and affective processing-related dopaminergic and serotonergic polymorphisms were genotyped (i.e., DRD2/ANKK1 TaqIA (rs1800497), dopamine receptor DRD4 exon-3 48 bp VNTR, and serotonin transporter linked polymorphic region 5-HTTLPR including the rs25531). Affectivity and ED were measured via parent- and/or self-report.

**Results:**

We first calculated bivariate correlations between polymorphisms, affectivity, and ED then compared the obtained (Fisher's *r* to *z*-transformed) values between with and without ADHD groups. There were no correlations that were significant – but several differed – across groups. In youth without ADHD, carrying the DRD2 rs1800497 T-allele was negatively associated both with negative affectivity (*p*_corr_=.033) and with self-rated ED (*p*_corr_=0.039). In youth with ADHD, carrying the DRD4 VNTR 7-repeat allele was positively associated with self-rated ED (*p*_corr_=0.008), and carrying the L'L’ relative to the low-expression S’ serotonergic allele was also positively associated with parent-rated ED (*p*_corr_=.042)

**Conclusion:**

Differences across with and without ADHD groups with regard to correlations between genetic polymorphisms - previously implicated in both ADHD and affective processing - and negative affectivity and emotion dysregulation indicate that certain genetic variants may differentially confer risk for affective outcomes, given ADHD status. These results have implications for targeted prevention of adolescent affective outcomes, which will be discussed during the presentation. That findings held across different indices of affective processing (dispositional affectivity and certain emotion dysregulation components) suggest these results may be robust.

